# Raghib Syndrome Presenting as a Cryptogenic Stroke: Role of Cardiac MRI in Accurate Diagnosis

**DOI:** 10.1155/2015/921247

**Published:** 2015-05-27

**Authors:** Vistasp J. Daruwalla, Keyur Parekh, Hassan Tahir, Jeremy D. Collins, James Carr

**Affiliations:** ^1^Conemaugh Memorial Hospital/Temple University, USA; ^2^Department of Cardiovascular Radiology, Northwestern University Feinberg School of Medicine, USA

## Abstract

Raghib Syndrome is a rare developmental complex, which consists of persistence of the left superior vena cava (PLSVC) along with coronary sinus ostial atresia and atrial septal defect. This Raghib complex anomaly has also been associated with other congenital malformations including ventricular septal defects, enlargement of the tricuspid annulus, and pulmonary stenosis. Our case demonstrates an isolated PLSVC draining into the left atrium along with coronary sinus atresia in a young patient presenting with cryptogenic stroke without the atrial septal defect. Majority of the cases reported in the literature were found to have the lesion during the postmortem evaluation or were characterized at angiography and/or echocardiography. We stress the importance of modern day imaging like the computed tomography (CT) angiography and cardiac MRI in diagnosis and surgical management of such rare lesions leading to cryptogenic strokes.

## 1. Introduction

Persistence of the left superior vena cava (PLSVC) is seen in 0.3%–0.5% of normal subjects [1, 2] and in up to 2.1%–4.3% of patients with congenital heart disease [3]. Coronary sinus ostial atresia with a PLSVC is usually associated with an anomalous connection between the coronary sinus ostium and the left atrium [4, 5]. Atresia of the coronary sinus ostium without this anomalous communication is rare since first described in 1738 [6]. Raghib syndrome is a rare developmental complex, which consists of PLSVC draining in the left atrium, coronary sinus ostial atresia, and atrial septal defect in the posteroinferior angle of the atrial septum. Okumori et al. stated that the atrial septal defect was true and this specific type of atrial septal defect associated with an absent coronary sinus [7]. Cases of an intact atrial septum and with PLSVC into left atrium were noted [8].

Due to the association with cardiac level right to left shunts, Raghib Syndrome has a significant risk of paradoxical embolization and is associated with varied degrees of reduced arterial blood oxygen saturation. Most of the cases reported in the literature were detected during postmortem evaluation or were characterized at angiography and/or echocardiography. We report a case of isolated PLSVC draining into the left atrium along with coronary sinus atresia in a young patient presenting with cryptogenic stroke evaluated with transthoracic echocardiography, computed tomography (CT), angiography and Cardiac MRI.

## 2. Case Presentation

The patient is a 31-year-old African-American, left handed female who was recently diagnosed with hypertension. She presented with left handed clumsiness, most notably in her left index finger and thumb, she also complains of an episode of slurred speech and left sided facial numbness that lasted for about 30 minutes to 1 hour. The patient was initially seen in a local hospital nearby and was reported to have an abnormal EKG with T wave inversions in leads v2–v4 and left ventricular hypertrophy but the patient refused admission and was admitted to our institution 10 days later. Her symptoms had improved two days after the attack but she still had difficulty with fine motor task like typing. During her stay she maintained a saturation of 95-96%.

Her neurological work-up included an MRI of the brain, which demonstrated a subacute infarct within the right precentral gyrus, involving the region of the hand knob correlating with her left hand weakness (Figure 5).

Transthoracic echocardiography revealed moderate left ventricular hypertrophy without regional wall motion abnormalities. The left atrium was mildly dilated. There was an increased mitral valve E point, ventricular septal separation; the visually estimated ejection fraction was 45–50%. There was no evidence of right to left shunting by agitated saline bubble contrast study performed in the right arm.

Her chest radiography demonstrated abnormal soft tissue densities along the right paratracheal region extending to the right main stem bronchus and along the right cardiomediastinal margin of unclear etiology which was further evaluated with a CT scan. CT imaging demonstrated persistence of the left superior vena cava draining into the left atrium without visualization of a coronary sinus. Cardiac venous drainage is seen directly into the inferior vena cava via the great cardiac vein. Subtle high attenuation contrast extends deep into the interventricular septum. While this may be a myocardial cleft in the same setting of possible left ventricular hypertrophy, the alternate possibility of a sinusoidal VSD is raised as a potential component of Raghib Syndrome. The mass seen in the chest radiograph along the cardiomediastinal margin was correlated to be a 5.4 cm pericardial cyst, and a 1 mm right middle lobe nodule was also noted.

**Figure 1:**
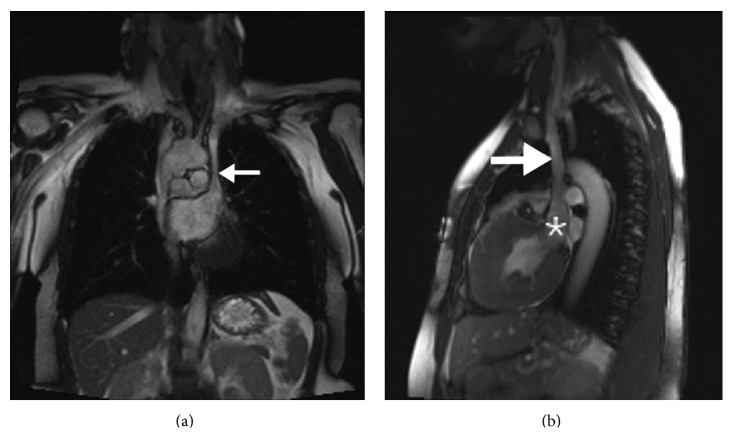
Coronal and sagittal T2 weighted images show a persistent left superior vena cava (arrow) draining in to the left atrial appendage (star).

For cryptogenic stroke, the patient underwent upper and lower extremity Doppler ultrasound as well as cardiac MRI. Doppler ultrasound showed no evidence of deep vein thrombosis. Cardiac MR demonstrated concentric hypertrophic cardiomyopathy, with relative sparing of the apical chamber. Left ventricular systolic function was moderately reduced with a calculated ejection fraction of 36%. The persistent left superior vena cava drained into the left atrium resulting in a right to left shunt with a *Qp*/*Qs* = 0.694 (Figure 1).

The patient's clinical diagnosis was Raghib syndrome with paradoxical embolization causing stroke. Although PLSVC serves as a right to left shunt, our patient during her stay has maintained oxygen saturation of above 90%. The right to left shunt caused by PLSVC is usually small and does not lead to significant oxygen desaturation. Transcatheter treatment is performed in patients with unroofed coronary sinus or an ASD and also seen in a reported case of LSVC which had a bridging with the RSVC by left brachiocephalic vein, such a treatment option was not considered in our patient as she did not have any of the above. She was discharged on aspirin, metoprolol, and a Holter monitor was placed prior to discharge. She will continue to follow up with the hospital for further evaluation 6 months later with a cardiac MRI.

## 3. Discussion

Raghib Syndrome is a rare cardiac anomaly that was originally described by Raghib et al. in 1965 as a developmental complex consisting of termination of the left superior vena cava in the left atrium, absence of the coronary sinus, and an atrial septal defect commonly located at the posterior-inferior angle of the atrial septum [9]. This complex was considered unique to Raghib Syndrome; however, cases with a normal atrial septum have been reported where the orifice of the unroofed coronary sinus functions as the interatrial communication [10].Our patient demonstrated an isolated persistent left superior vena cava draining into the left atrium along with an absence of the coronary sinus (Figure 6) and absence of ASD (Figure 7). She also has hypertrophic cardiomyopathy leading to ventricular dysfunction; association of this condition with Raghib syndrome has not been reported before.

Embryologically, persistence of the left superior vena cava (PLSVC) occurs due to failure of involution of the left horn of the embryonic sinus venosus and is one the most common venous anomalies of the chest. Drainage of the PLSVC into the left atrium due to congenital defects which prevent the rotation of the sinoatrial region; this in turn causes the left and the right cardinal veins to lie at the same level rather than the usual inferior location of the right cardinal vein, thus blocking the development of the coronary sinus. As a result a PLSVC draining into left atrium is commonly associated with coronary sinus ostial atresia [7]. LSVC may serve as a collateral channel secondary to coronary sinus atresia. This anatomic configuration is vital to recognize at open heart surgery so that the cardiac venous return can be identified and preserved. Retrograde administration of cardioplegia is also relatively contraindicated. The coronary sinus catheter balloon may not be able to occlude the dilated coronary sinus, resulting in failure to ensure retrograde flow of cardioplegia to the myocardium. Also, cardioplegia delivered would largely be distributed to the left internal jugular and left subclavian veins, rather than myocardium [11, 12]. Careful mapping of the coronary venous anatomy and dissection of the coronary sinus are necessary before reanastomoses of the PLSVC into the right atrium is attempted [11].

**Figure 2:**
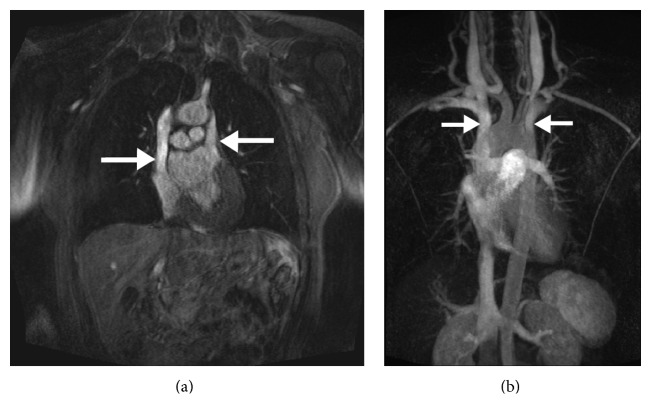
Static and three-dimensional MR angiography confirms the presence of right and left superior vena cava (arrows) draining in to the corresponding atrial chambers leading to a right-to-left shunt.

**Figure 3:**
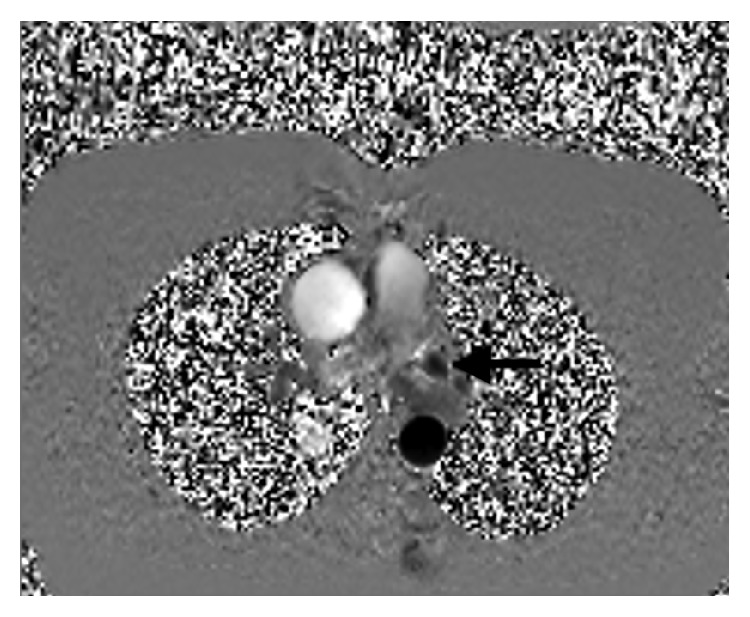
Axial 2D phase contrast images in the upper chest show caudally directed flow in the left superior vena cava (arrow).

**Figure 4:**
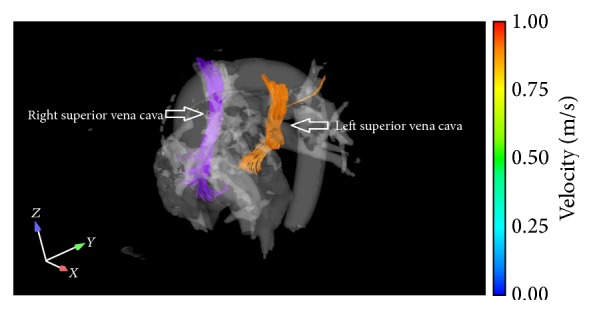
Whole heart 4D flow image demonstrating differential flow into the atria. Flow from the right superior vena cava (SVC) to the right atrium and left superior vena cava to the left atrium.

**Figure 5:**
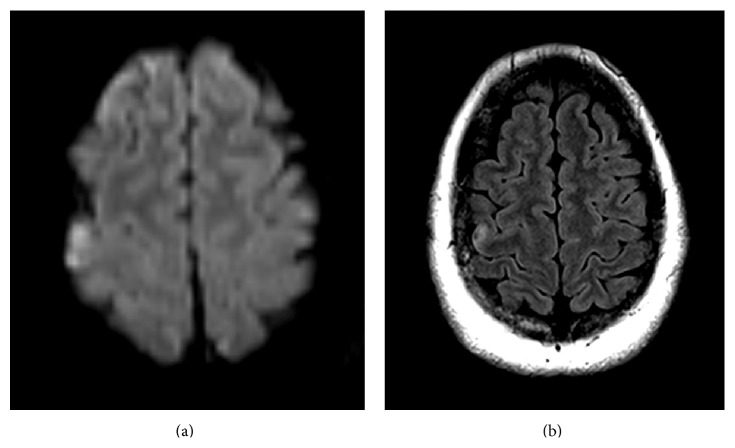
Restricted diffusion on DWI with increase T2 signal on FLAIR and postcontrast enhancement in right frontal lobe is consistent with subacute infarct within the right precentral gyrus.

**Figure 6:**
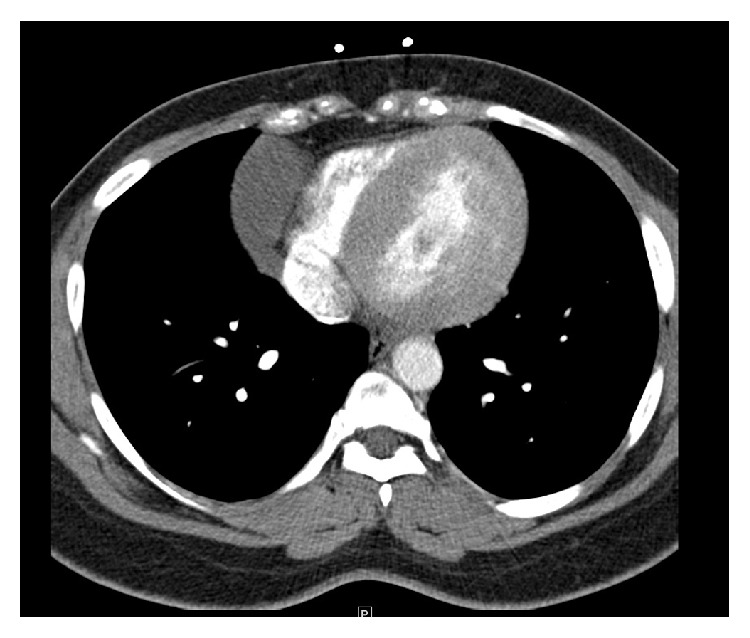
Coronary sinus atresia: absence of coronary sinus at the expected location on axial contrast CT chest image.

**Figure 7:**
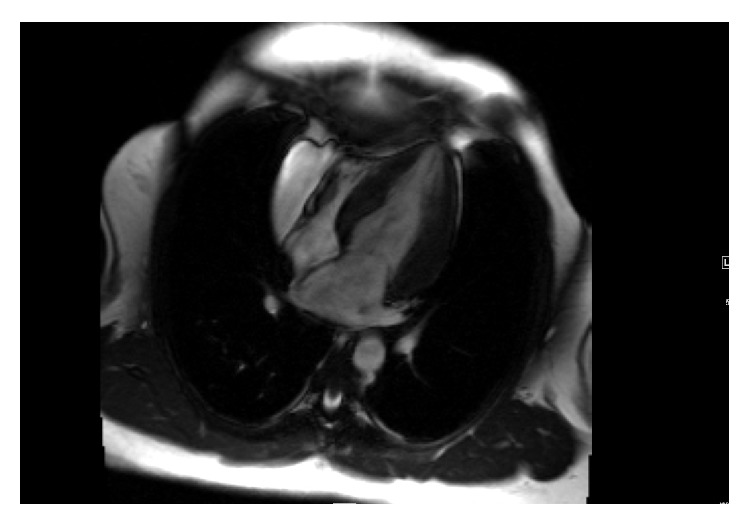
Four chamber steady-state free precession imaging shows intact interatrial septum. No ASD noted.

Precise anatomical detailing and multiresolution image building capacity of cardiac CT scan is of high yield in such cases especially in the plane of the atrioventricular groove in a short axis view. The shunt between the left atrium and the coronary sinus can be easily detected on a cardiac CT scan by insertion of contrast in the left arm vein and noting its presence in the left atrium [13]. MRI on the other hand with its phase contrast cine images may demonstrate turbulent flow from left atrium to coronary sinus, thus providing a distinctive advantage in detecting left to right shunt and estimating the ratio of pulmonary to systemic flow (Figure 3). The 3D images of cardiac CT scan and MRI not only facilitate in diagnosing the anomaly but also play an important role in planning surgical interventions like closure of PLSVC with coils or coronary sinus stent placement (Figure 2) [14, 15]. A novel cardiac MRI 4D flow technique, still under research phase, displays differential flow into the atria (Figure 4). Overall cardiac Ct scan and MRI provide an unparalleled edge in visualization of such rare developmental anomalies and assist in prevention of paradoxical cerebral embolization and abscess formation [16].

## 4. Conclusion

Raghib Syndrome is considered to be a benign anomaly yet is associated with paradoxical embolization, diminished arterial oxygen saturation, and has implications for cardiac surgery as well as considerations for the location of intravenous access. Cardiac MRI and cardiac CT are useful imaging modalities in the evaluation of anomalies of the systemic venous return, enabling quantification of the flow disturbance and characterization of the anatomy.

## References

[1] Biffi M., Boriani G., Frabetti L., Bronzetti G., Branzi A. (2001). Left superior vena cava persistence in patients undergoing pacemaker or cardioverter-defibrillator implantation: a 10-year experience. *Chest*.

[2] Perloff J. K. (1994). Congenital anomalies of vena caval connection. *The Clinical Recognition of Congenital Heart Disease*.

[3] Horrow J. C., Lingaraju N. (1989). Unexpected persistent left superior vena cava: diagnostic clues during monitoring. *Journal of Cardiothoracic Anesthesia*.

[4] Mantini E., Grondin C. M., Lillehei C. W., Edwards J. E. (1966). Congenital anomalies involving the coronary sinus. *Circulation*.

[5] de Leval M. R., Ritter D. G., McGoon D. C., Danielson G. K. (1975). Anomalous systemic venous connection: surgical considerations. *Mayo Clinic Proceedings*.

[6] Le Cat (1738). *Histoire de l'Acad Royale des Sciences*.

[7] Okumori M., Hyuga M., Ogata S., Akamatsu T., Otomi S., Ota S. (1982). Raghib's syndrome: a report of two cases. *Japanese Journal of Surgery*.

[8] Goor D. A., Lillehei C. W. (1975). *Congenital Malformations of the Heart: Embryology, Anatomy, and Operative Considerations*.

[9] Raghib G., Ruttenberg H. D., Anderson R. C., Amplatz K., Adams P., Edwards J. E. (1965). Termination of left superior vena cava in left atrium, atrial septal defect, and absence of coronary sinus. *Circulation*.

[10] Allen H. D., Driscoll D. J., Shaddy R. E., Feltes T. F. (2012). *Moss and Adam's Heart Disease in Infants, Children and Adolescent*.

[11] Nsah E. N., Moore G. W., Hutchins G. M. (1991). Pathogenesis of persistent left superior vena cava with a coronary sinus connection. *Pediatric Pathology*.

[12] Paval J., Nayak S. (2007). A persistent left superior vena cava. *Singapore Medical Journal*.

[13] Brancaccio G., Miraldi F., Ventriglia F., Michielon G., di Donato R. M., de Santis M. (2003). Multidetector-row helical computed tomography imaging of unroofed coronary sinus. *International Journal of Cardiology*.

[14] Hahm J. K., Park Y. W., Lee J. K. (2000). Magnetic resonance imaging of unroofed coronary sinus: three cases. *Pediatric Cardiology*.

[15] Choe Y. H., Kang I.-S., Park S. W., Lee H. J. (2001). MR imaging of congenital heart disease in adolescents and adults. *Korean Journal of Radiology*.

[16] Kim E. M., Moore P. B., Singh S. P., Lloyd S. G. (2011). Cardiovascular magnetic resonance imaging of the Raghib complex. *Journal of the American College of Cardiology*.

